# Zoonotic Risk of Intestinal Parasites in Ghana′s Protected Areas: A Nexus for Human–Nonhuman Primates–Dog Interactions

**DOI:** 10.1155/japr/4239782

**Published:** 2026-05-16

**Authors:** Sandra S. Gyarteng Mensah, Brian Acheampong, Adrian Streit, John A. Larbi

**Affiliations:** ^1^ Department for Theoretical and Applied Biology, Kwame Nkrumah University of Science and Technology, Kumasi, Ghana, knust.edu.gh; ^2^ Department of Integrative Evolutionary Biology, Max Planck Institute for Biology, Tübingen, Germany; ^3^ Department of Pharmaceutical Sciences, Sunyani Technical University, Sunyani, Ghana, stu.edu.gh; ^4^ Department of Microbiology and Immunology, State University of New York Upstate Medical University, Syracuse, New York, USA, upstate.edu

**Keywords:** dogs, humans, intestinal parasites, nonhuman primates, One Health, zoonotic disease

## Abstract

Intestinal parasites with zoonotic potential and the risk factors associated with their transmission pose a significant health threat to humans, nonhuman primates, and dogs living in or around protected areas. This study is aimed at determining the prevalence, species richness, and associated risk factors of the interaction between humans, nonhuman primates, and dogs in Boabeng Fiema, Mole National Park, and Shai Hills reserve resources. A cross‐sectional study was used to collect stool samples and questionnaire data. The stool was analyzed using a formal ether sedimentation method, and a stratified questionnaire was used to assess the risk factors associated with the interaction between the three hosts in and around the three protected areas. Using R software, the chi‐square test was used to evaluate the significance of associations with dog management, hygiene, and sanitation practices. Prevalence in humans was 10.56% in Boabeng Fiema, 44.94% in Mole National Park, and 58.13% in Shai Hills; dogs and nonhuman primates carried higher burdens (dogs: 89.59%, 82.14%, and 27.27%; nonhuman primates: 60.78%, 42.85%, and 47.72%, respectively). Hookworms and ascarids were the common parasites shared among all three hosts. Shai Hills recorded the highest species richness. Parasites identified in the study were strongylids, hookworms, *Ascaris* sp., *Balantidium* sp., *Diphyllobothrium* sp., *Entamoeba* sp., *Schistosoma* sp., *Trichuris* sp., *Taenia* sp., and *Hymenolepis* sp. Poor dog confinement, open defecation, inconsistent wearing of footwear, and suboptimal hygienic and deworming practices contributed to sustaining transmission. The high prevalence of intestinal parasites recorded in dogs and nonhuman primates should be treated as a public health threat. These results emphasise the urgent need to integrate intervention, using a One Health approach to minimise the zoonotic transmission of intestinal parasites in and around Ghana′s protected areas.

## 1. Introduction

Zoonoses are infectious diseases transmitted naturally from wildlife or domestic animals to humans, and they represent a significant public health concern worldwide [1, 2]. Within this context, it is important to distinguish between pathogens of zoonotic evolutionary origin and ongoing zoonotic infections. Pathogens with zoonotic evolutionary origin are those that arose from a historical host‐switch event and subsequently adapted to human‐to‐human transmission, whereas zoonotic infections are those in humans that arose through continual spillover from animal reservoirs [3].

About 60% of emerging infectious diseases are zoonotic in origin, and their control is challenging across developed and developing regions [4]. This challenge is greater because zoonotic diseases originate from a broad spectrum of etiological agents such as bacteria, fungi, viruses, and parasites, each with different modes of transmission, environmental persistence and host range. Such diversity complicates surveillance and control strategies, as interventions effective against one class of pathogens may be ineffective for others [5].

Among the various types of zoonotic agents, parasites are concerning because of their complex life cycles, often involving multiple host species and stages of development that allow them to persist in the environment for an extended period outside the host [6, 7]. Their ability to adapt biologically helps them survive and increases their potential for transmission across species. While bacterial and viral zoonoses often dominate public health discussions and policy making, parasitic zoonoses, especially intestinal parasite infections (IPIs), tend to receive less attention despite their epidemiological relevance [8, 9].

Globally, intestinal parasitic infections affect an estimated 3.5 billion people, causing 200,000 deaths annually [10, 11]. The impact is greater in low‐ and middle‐income countries, where infrastructural, environmental, and socioeconomic factors create conditions favorable for parasite transmission. More so, epidemiological studies linked the transmission of strongyloidiasis, amoebiasis, hookworm infections, and other IPIs to poor sanitation, contaminated food or water, poor hygiene, low level of education, socioeconomic factors and population growth [12]. Significantly, close human interaction with dogs and nonhuman primates (NHPs) through shared environment or proximity to human habitation increases the likelihood of opportunities for cross‐species transmission of zoonotic parasites [13]. These animals can serve as reservoirs and incidental hosts, sustaining parasite populations in shared environments.

A systematic review by Barnes et al. [13] showed that *Strongyloides* sp., ascarids, hookworm, *Entamoeba*, *Balantidium* sp., and so on are among the parasites with zoonotic potential. Evidence from experimental studies reinforces this concern. For example, *S. stercoralis* has been successfully transmitted from humans to dogs [14, 15]. Also, based on the nuclear 18Sr DNA, the mitochondrial *cox-1* locus, and whole genome sequence, it was found that at least two populations of *S. stercoralis* exist in dogs, only one of which appears to be shared with humans [16–19]. In Ghana, owned and stray dogs are permitted to roam freely, increasing their exposure to contaminated environments and other infected hosts (Johnson et al., 2015) [20]. Recent studies by Anim‐Baidoo et al. [21] recorded 38.2% of IPIs in dogs in Accra, with community dogs bearing exceptionally high infection rates (70.8%), including helminth parasites such as hookworms, *Toxocara* spp., and taeniids. Despite this, limited research examines zoonotic parasites in dogs or their interactions with humans in the country. Studies have shown that dogs can carry *S. stercoralis* genotypes that are infectious to humans, implying that similar dynamics may exist in Ghana [17–19]. This is particularly concerning in protected areas where humans, dogs, and wildlife regularly interact.

NHPs are also at a greater risk of sharing parasites because of their close phylogenetic relationship to humans. Their frequent migration between human settlements and forest areas in quest of food leads to more opportunities for parasite exchange in many African contexts [22]. In rural African communities and regions bordering wildlife reserves, the ecological overlap between humans, wildlife, and domestic animals is intensified by population growth, agricultural expansion, and habitat encroachment [23, 24]. Such proximity facilitates the exchange of parasites and the emergence of novel transmission cycles. In these communities, dog owners who are into farming and hunting frequently take their dogs to forested and protected areas, where they can be exposed to wildlife parasites and transmit these pathogens back into human communities. NHPs may also act as bridging hosts, contaminating the environment with parasite eggs or cysts that infect humans and domestic animals.

Protected areas are crucial for biodiversity conservation, but can also act as hotspots for zoonotic transmission due to the convergence of humans, domestic animals, and wildlife. Farming, tourism, and resource gathering bring communities and their dogs into close contact with wildlife habitats. In such areas, dogs can serve as potential carriers for the transmission of parasites, whereas NHPs pose additional risks due to their biological similarity to humans [25, 26]. Despite the potential public health threat, limited data exist on the prevalence and diversity of intestinal parasites in humans, dogs, and NHPs in Ghana′s protected areas. Without such data, developing effective public health strategies to address zoonotic IPIs is challenging. In light of the above, this study determined the prevalence and species composition of intestinal parasites in humans, domestic dogs, and NHPs in three protected areas in Ghana. It also accessed the risk factors associated with human–dog–NHP interactions and hygienic practices. Using a One Health approach, the study goal was to offer practical guidance for measures that prevent cross‐species transmission and protect community health.

## 2. Materials and Methods

### 2.1. Study Area

**Figure 1:**
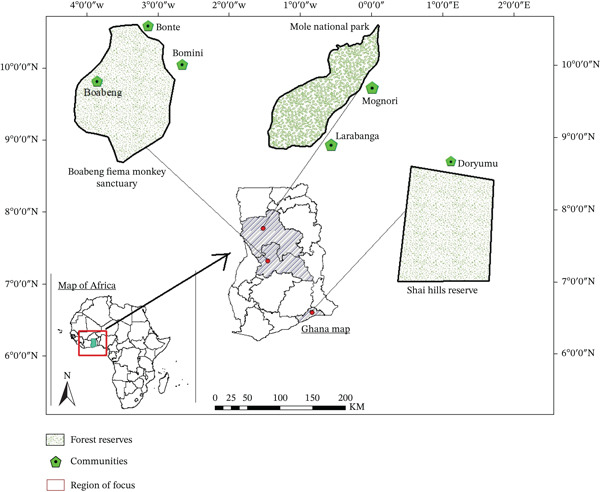
Map of Ghana showing the three study sites.

The study was conducted in Ghana′s Boabeng‐Fiema Monkey Sanctuary, Mole National Park, and Shai Hills Resource Reserve (Figure 1). These three places were selected due to their unique ecological characteristics, rich biodiversity, and the close interaction between humans, domestic animals, and wildlife. These three sites are located in different ecological zones of Ghana, which makes them well suited for researching the dynamics of zoonotic intestinal parasitic infections in different ecological zones. In each site, the protected area and fringe communities constituted sampling locations.

### 2.2. Boabeng‐Fiema Monkey Sanctuary

Boabeng‐Fiema Monkey Sanctuary is a dry semi‐deciduous forest located in the forest–savanna transition zone in the middlebelt of Ghana. It lies within latitudes 7°40 ^′^00 ^″^N to 7°44 ^′^10 ^″^N and longitudes 1°37 ^′^45 ^″^W to 1°42 ^′^00 ^″^W in the Nkoranza North district of the Bono East Region [27, 28]. This study area is a sacred grove traditionally protected due to its cultural and spiritual significance, which has contributed to the wildlife conservation efforts [29]. In addition to its ecological importance, the sanctuary also serves as tourist destination. The Boabeng‐Fiema Monkey Sanctuary is home to two species of NHPs: *Colobus vellerosus* (black and white colobus) and *Cercopithecus campbelli lowei* (Mona monkeys). A study conducted by Wiafe et al. (2023) [30] reported 602 individuals of *C. vellerosus* distributed across 34 groups and 315 individuals of *C. campbelli lowei* in 26 groups. These groups are spread in six surrounding communities, three of which were used for the study: Boabeng (7°43 ^′^06.5 ^″^N 1°41 ^′^36.4 ^″^W), Bonte (7°44 ^′^26.6 ^″^N 1°40 ^′^07.5 ^″^W), and Bomini (7°43 ^′^37.3 ^″^N 1°39 ^′^19.1 ^″^W). The inhabitants of these areas are primarily farmers; they cultivate maize, cashew, yams, and cassava. Notably, although owning dogs is officially not allowed in some of these communities due to government regulations, some residents in Bonte and Bomini still keep them [28].

### 2.3. Mole National Park

Mole National Park is the largest national park with the widest wildlife range in Ghana spanning an area of 4480 km^2^. The park is located in the Guinea Savannah woodland in northern Ghana within the latitudes 09°12 ^′^00 ^″^N to 10°06 ^′^00 ^″^N and longitudes 01°25 ^′^00 ^″^W to 02°17 ^′^00 ^″^W [28, 31]. In Mole, one will see elephants, antelopes, bushbucks, monkeys, warthogs, baboons, patas monkey, green (vervet) monkey, and other smaller wildlife. Baboons are frequently seen in human areas, making them potential reservoirs for zoonotic parasites and other pathogens (Ryan et al. 2012) [32]. In addition, some people in the nearby communities are farmers and hunters. Most of these hunters and farmers are seen with dogs when hunting or farming. This study was conducted in two rural communities surrounding the park: Laranbaga (9°13 ^′^12.6 ^″^N 1°51 ^′^30.6 ^″^W) and Mognori (9°17 ^′^23.5 ^″^N 1°46 ^′^25.0 ^″^W). Even though dog ownership, settlements, and livestock grazing are restricted within the park boundaries, human–wildlife interactions still occur, especially in buffer zones.

### 2.4. Shai Hills

Shai Hills Reserve is a small fenced area of about 51 km^2^. The vegetation is a coastal savannah type with high grassland and low dry forest that lies between latitudes 5°50 ^′^00 ^″^N to 5°56 ^′^00 ^″^N and longitudes 0°01 ^′^00 ^″^E to 0°06 ^′^00 ^″^E in the Greater Accra region [28, 33]. Shai Hills is known for hiking, cave exploration, rock climbing, and other ecotourism activities, which contribute to conservation and livelihoods for locals. The Major wildlife are olive baboons, green monkeys, cobs, and bushbucks. In this reserve, the indigenous people go to the baboon‐dominated mountains every year to pay homage to their ancestors and interact with the baboons as pets, thereby presenting opportunities for interaction [28, 34]. The study was conducted in the reserve area and in the Doryumu community (5°53 ^′^45.8 ^″^N 0°01 ^′^05.9 ^″^E). People living in the Doryumu community engage in petty trading and subsistence farming.

### 2.5. Study Design and Sample Collection

A cross‐sectional study design was conducted to collect stool samples from humans, dogs, and NHPs and to interview community members in study locations.

### 2.6. Ethical Clearance Consideration

Ethical clearance for the study was sought and obtained from the Committee for Human Research Publication and Ethics of the School of Medical Sciences, Kwame Nkrumah University of Science and Technology (Ref. Nos.: CHRPE/AP/278/22 and CHRPE/AP/678/22). A written informed consent was secured from all human participants prior to sample collection. In addition, approval was obtained from the Wildlife Division of the Forestry Commission of Ghana (Ref. No.: WD/A30/VOL.13/10) ([28]).

### 2.7. Stool Sample Collection: Humans, Dogs, and Primates

Community members in and around the reserve areas who consented to participate were given sterile stool containers labeled with their identification number and the date of collection. They were also given disposable gloves to avoid direct contact with fecal matter. Participants were carefully guided through the sample collection procedure to ensure the samples were not contaminated by soil. Additionally, stool samples were collected from the rectum of dogs with the assistance of a licensed veterinary professional to ensure sample freshness and avoid soil contamination. In each of the reserve areas, experienced reserve staff assisted in identifying and trailing different primate colonies within the study areas; samples were collected systematically following each colony for 3 h before rotating to another colony [28]. Only the inner portion of the fecal matter was extracted using sterile tools to avoid any external contamination from the soil or other environmental materials. A total of 2 g of fresh stool samples from humans, dogs, and NHPs were used for the Formalin Ethyl Sedimentation Method.

### 2.8. Laboratory Analysis

Using the Formalin Ethyl Sedimentation Method, 2 g of stool samples were emulsified in 10% formal saline. The emulsion was filtered into a 15‐mL centrifuge tube using a gauze placed in a funnel to the 7‐mL mark. Approximately 3 mL of ethyl acetate was added to reach 10 mL. This was centrifuged at 1500 rpm for 5 min. The supernatant was discarded after settling. Sediments were examined microscopically at 10× and 40× magnifications to detect parasitic eggs, cysts, and larvae [35].

### 2.9. Questionnaire Administration

A structured questionnaire (Supporting File 1) was administered to participants to determine the risk factors associated with interactions between humans, dogs, and NHPs. Participants were assisted in answering the questionnaire by translating and also defining terminologies in their local dialect.

### 2.10. Data Analysis

Questionnaire and microscopic data were organised and entered into an Excel spreadsheet. Chi‐square tests were used to evaluate the significance of associations with dog/primate management, hygiene, and sanitation practices.

## 3. Results

### 3.1. Prevalence of Intestinal Parasites Across Study Locations

Five hundred four stool samples were collected across the three study areas: Boabeng Fiema, Mole National Park, and Shai Hills. Of these, 274 were from humans, 87 from dogs, and 143 from NHPs. At Boabeng Fiema, 15 (10.56%) of human, 43 (89.58%) dogs, and 31 (60.78%) NHPs samples showed IPIs (Table 1). Also, in Mole National Park, 40 (44.94%) humans, 23 (82.14%) dogs, and 21(42.85%) NHPs had IPIs. In Shai Hills, 25 (58.14%) humans, 3 (27.27%) dogs, and 21(47.72%) NHPs had intestinal parasites. Overall, dog (79.31%) samples recorded the highest prevalence of IPIs, followed by NHPs (50.69%) and humans (29.20%).

**Table 1:** Overall prevalence of intestinal parasitic infection in humans, dogs, and nonhuman primates across three study areas.

Study area	Humans		Dogs		Primates	
	Infected (%)	Not infected (%)	Infected (%)	Not infected (%)	Infected (%)	Not infected (%)
Boabeng Fiema	15 (10.56%)	127 (89.44%)	43 (89.58%)	5 (10.41%)	31 (60.78%)	20 (39.16%)
Mole National Park	40 (44.94%)	49 (55.06%)	23 (82.14%)	5 (17.86%)	21 (42.85%)	28 (57.14%)
Shai Hills	25 (58.14%)	18 (41.86%)	3 (27.27%)	8 (72.73%)	21 (47.72%)	23 (52.27%)
Total	80 (29.20%)	194 (70.80%)	69 (79.31%)	18 (20.69%)	73 (50.69%)	70 (49.31%)

### 3.2. Prevalence and Diversity of Intestinal Parasites in Humans, Mona Monkey, Black and White Colobus Monkey, and Dogs in Boabeng Fiema

**Figure 2:**
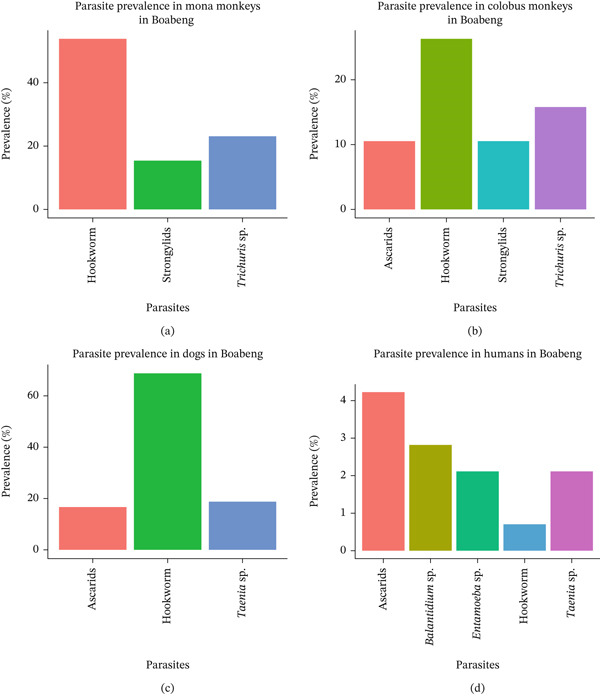
Prevalence and species richness of intestinal parasites in all three hosts at Boabeng‐Fiema Monkey Sanctuary. (a) Parasite prevalence in mona monkeys in Boabeng; (b) parasite prevalence in colobus monkeys in Boabeng; (c) parasite prevalence in dogs in Boabeng; and (d) parasite prevalence in humans in Boabeng.

The prevalence of hookworms in mona monkeys at Boabeng Fiema was above 45%. This was followed by *Trichuris* sp., with a prevalence slightly above 20% (Figure 2). *Strongylids* have the lowest recorded prevalence, infecting less than 15% of individuals. Also, hookworms were recorded to have the highest prevalence in colobus monkeys, close to 25%. *Trichuris* sp. followed with about 15% prevalence. Both ascarids and *Strongylids* showed similar prevalence levels, both around 10%. The prevalence of hookworms in dogs at Boabeng Fiema was extremely high, exceeding 65%. This was followed by ascarids and *Taenia* sp. with 15% prevalence. Ascarids (4.8%) were the most prevalent parasites recorded in humans, followed *by Balantidium* sp., *Entamoeba* sp., *Taenia* sp., and hookworm sp., with prevalence of 2.8%, 2.3%, 0.8%, and 2.3%, respectively. In addition, species richness in Boabeng Fiema was low with seven species of parasites identified.

### 3.3. Prevalence and Diversity of Intestinal Parasites in Humans, Baboons, Patas Monkey, and Dogs in Mole National Park

**Figure 3:**
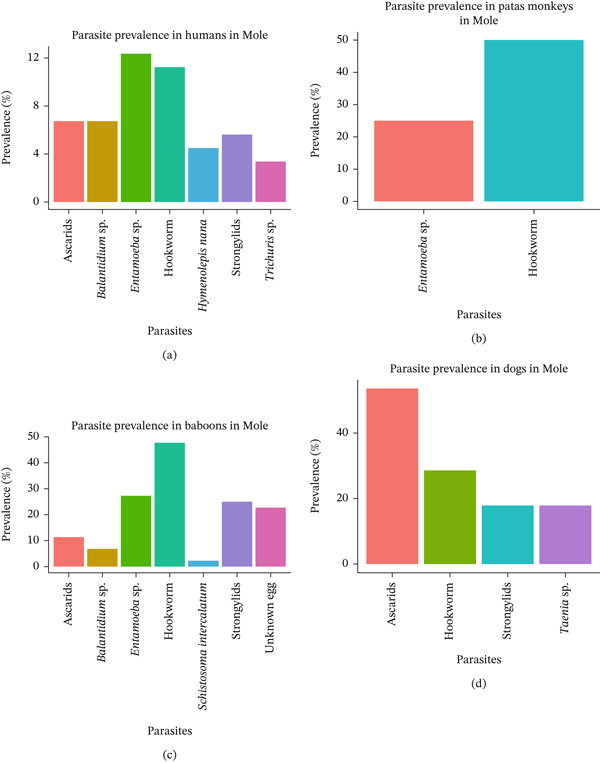
Prevalence and species richness of intestinal parasites in all three hosts at Mole National Park. (a) Parasite prevalence in humans in Mole; (b) parasite prevalence in patas monkeys in Mole; (c) parasite prevalence in baboons in Mole; and (d) parasite prevalence in dogs in Mole.

In humans (Figure 3a), *Entamoeba* sp. recorded the highest prevalence, affecting approximately 12% of participants. This was followed by hookworm infections, which were above 10%. Prevalence for ascarids, *Balantidium* sp., *Strongylids*, and *Hymenolepis* sp. was around 5%–7%. *Trichuris* sp. was the lowest prevalence recorded among human participants, under 5%. In patas monkeys (Figure 3b), only two parasites were detected: *Entamoeba* sp. and hookworms with a prevalence of 48% and 22%, respectively. Hookworms were the most prevalent parasite, with 40% of the baboons (Figure 3c) infected, followed by *Entamoeba* sp. (20%). Ascarids, *Balantidium* sp., *Strongylids*, and some unidentified eggs occurred at relatively lower prevalence. In dogs (Figure 3d), ascarids recorded the highest prevalence at 45% followed by hookworm, with 25% of the dog population infected. *Strongylids* and *Taenia* sp. They were also identified as having a prevalence of 15% and 20%, respectively. Ten different parasite species were recorded, indicating high species richness in Mole National Park.

### 3.4. Prevalence and Diversity of Intestinal Parasites in Humans, Baboons, and Dogs in Shai Hills

**Figure 4:**
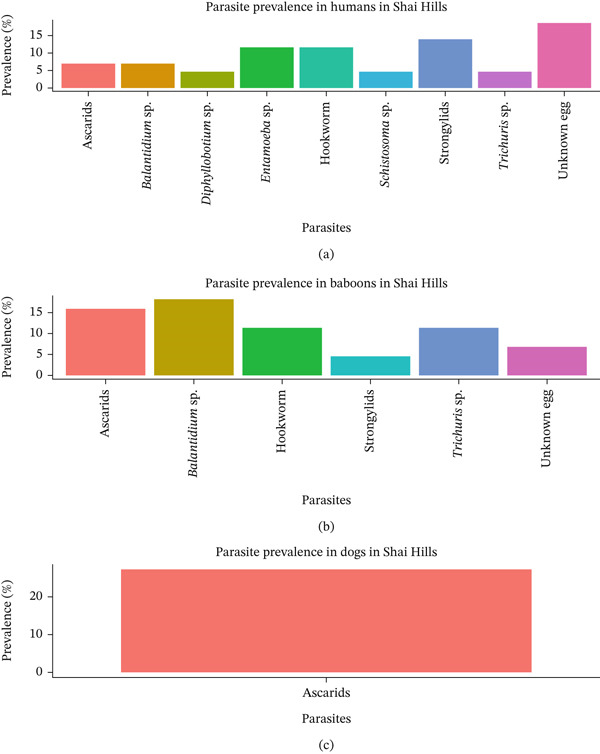
Prevalence and species richness of intestinal parasites in all three hosts at Shai Hills. (a) Parasite prevalence in humans in Shai Hills; (b) parasite prevalence in baboons in Shai Hills; and (c) parasite prevalence in dogs in Shai Hills.

In humans, unidentified eggs recorded the highest prevalence, followed by *Strongylid*s and hookworms (Figure 4). Ascarids, *Balantidium* sp., *Diphyllobothrium* sp., *Entamoeba* sp., *Schistosoma* sp., and *Trichuris* sp. were also identified but at relatively lower prevalence levels, generally under 15%. In baboons (NHPs), ascarids, and *Balantidium* sp. showed the highest prevalence rates, each around 15%, followed by *Trichuris* sp. and hookworms. *Strongylids* and an unknown egg type were detected at lower frequencies. In dogs, only ascarids were detected, showing a high prevalence of 25%. Generally, ascarids were commonly detected across all three host groups. Humans showed the most diversity of parasite types, whereas dogs had the least, with only ascarids identified. Among the NHPs, significant infections were observed with both ascarids and *Balantidium* sp. In Shai Hills, nine different species were identified.

### 3.5. Common Parasites Shared Among Humans, Dogs, and NHPs in Boabeng Fiema, Mole National Park, and Shai Hills

**Figure 5:**
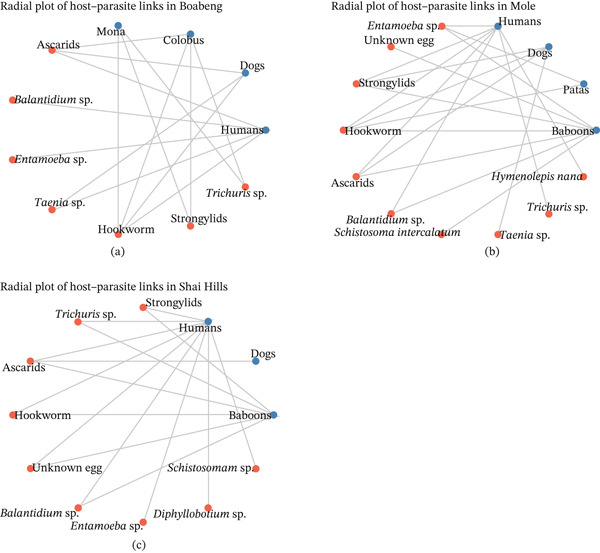
Intestinal parasites (red color) distribution among humans, nonhuman primates, and dogs (blue color) in (a) Boabeng‐Fiema Monkey Sanctuary, (b) Mole National Park, and (c) Shai Hills Resource Reserve.

In Boabeng Fiema, ascarids and hookworms were parasites shared among humans, dogs, and NHPs (Figure 5a). The parasites shared among dogs, humans, and NHPs were hookworm, *Strongylids*, and ascarids in Mole (Figure 5b). Also, in Shai Hills, the common parasites shared among dogs, NHPs, and humans were ascarids. Additionally, hookworms were present in both humans and NHPs (Figure 5c).

### 3.6. Dog Management Practices and Human–Dog Interactions in Boabeng and Shai Hills

In the Boabeng‐Fiema Monkey Sanctuary, distribution of respondents among categories of all the dog‐related variables, including the number of dogs owned, years of ownership, purpose for keeping dogs (companionship, hunting, security, sale, and meat), dog‐keeping style, feeding methods, and stool disposal practices were statistically different (*p* < 0.05; Table 2). In contrast, at the Shai Hills Resource Reserve, the only significant variation was dog ownership status (*p* = 0.0016), with all other dog‐related variables showing no statistically significant differences (*p* > 0.05).

**Table 2:** Comparison of dog management practices and human–dog interactions in Boabeng and Shai Hills.

Variable	Boabeng‐Fiema Monkey Sanctuary				Shai Hills Resource Reserve			
	Proportions	Chi‐square	df	*p* value	Proportions	Chi‐square	df	*p* value
Dog ownership								
Yes	1	—	—	—	0.207	9.9655	1	0.001595
No	0				0.793			
Number of dogs								
1–2 dogs	0.583	46.55	2	< 0.001	0.833	2.6667	1	0.1025
3–5 dogs	0.342				0.167			
6–10 dogs	0.075				0			
Years of dog ownership								
< 1 year	0.248	9.5455	3	0.02285	0.833	2.6667	1	0.1025
1–2 years	0.347				0.167			
3–4 years	0.256				0			
> 5 years	0.149				0			
Purpose for dogs								
Companionship								
Yes	0.183	48.133	1	< 0.001	0.333	0.66667	1	0.4142
No	0.817				0.667			
Hunting								
Yes	0.361	9.1513	1	< 0.001	0	—	—	—
No	0.639				1			
Security								
Yes	0.874	66.563	1	< 0.001	0.167	2.6667	1	0.1025
No	0.126				0.833			
Commercial sale								
Yes	0.017	111.13	1	< 0.001	0	—	—	—
No	0.983				1			
Food (meat)								
Yes	0.008	118.03	1	< 0.001	0	—	—	—
No	0.992				0	—	—	—
Dog‐keeping behavior								
Confined	0	26.851	1	< 0.001	0.167	1	2	0.6065
Semiconfined	0.264				0.500			
Not confined	0.736				0.333			
Dog‐feeding behavior								
On the floor	0.697	138.52	3	< 0.001	0.167	1	2	0.6065
In a bowl	0.197				0			
Outside home	0.008				0.333			
Sometimes in a bowl	0.098				0.500			
Dog‐feeding in a bowl								
Always	0.333	12.25	2	0.002187	0	—	—	—
Never	0.042				0			
Sometimes	0.625				0			
Dog stool handwash								
Always	0.067	77.6	2	< 0.001	0.167	1	2	0.6065
Never	0.700				0.333			
Sometimes	0.233				0.500			
Dog stool disposal								
Accessible	0.944	28.444	1	< 0.001	0.75	1	1	0.3173
Not accessible	0.056				0.25			

### 3.7. Direct and Indirect Human–NHP Interactions in Boabeng Fiema and Shai Hills

In Boabeng Fiema, the nature of interaction between respondents and NHPs showed important differences: Respondents who confirmed proximity to primates, direct contact with primates, direct hand contact with primates, direct contact with primate stool as well as those who observed primate contact with soil and water were significantly lower than those who did not (*p* < 0.030; Table 3). A significantly higher number of respondents had direct contact with primates in the dry season (*p* = 0.001). Most respondents reported that they always washed their hands after direct contact with primate stool. This was statistically different from respondent subgroups who sometimes washed their hands and those who did not wash their hands at all (*p* < 0.001). Specific type of hand washing behavior differed significantly among respondents. Most respondents always washed their hands with soap and water after contact with primate, followed by sometimes with soap and water, water only, and sometimes with water only. In the Shai Hills Resource Reserve, respondents who had proximity to primates and direct primate contact were also significantly lower than those who did not (*p* = 0.002). People who sometimes washed their hands after direct contact with primates were significantly higher than those who did not wash hands at all or those who always washed their hands (*p* = 0.011).

**Table 3:** Humans–nonhuman primates and hygienic practices at Boabeng Fiema and Shai Hills.

Variable	Boabeng‐Fiema Monkey Sanctuary				Shai Hills Resource Reserve			
	Proportions	Chi‐square	df	*p* value	Proportions	Chi‐square	df	*p* value
Nonhuman primate around household								
Yes	0.221	37.902	1	< 0.001	0.207	104.94	3	0.002
No	0.779				0.793			
Ever had contact with nonhuman primate								
Yes	0.18	49.869	1	< 0.001	0.214	9.1429	1	0.002
No	0.82				0.786			
Nonhuman primate contact frequency								
Less frequently	0.273	2.3636	3	0.500				
Daily	0.227							
Weekly	0.136							
Monthly	0.364							
Nonhuman primate contact with hands								
Yes	0.1	12.8	1	< 0.001	0.2	1.8	1	0.179
No	0.9				0.8			
Nonhuman primate contact to soil around household								
Yes	0.25	5	1	0.025	0.4	0.2	1	0.655
No	0.75				0.6			
Nonhuman primates contact to water in household								
Yes	0.25	5	1	0.025				
No	075							
Nonhuman primates contact to food in household								
Yes	0.5	0	1	1	0.2	1.8	1	0.179
No	0.5				0.8			
Nonhuman primates contact in rain season								
Yes	0.318	2.9091	1	0.088				
No	0.682							
Nonhuman primates contact in dry season								
Yes	0.864	11.636	1	0.001	0.5	0	1	1.000
No	0.136				0.5			
Contact to primate stool								
Yes	0.182	8.9091	1	0.003	0.333	0.66667	1	0.414
No	0.818				0.667			
Handwash after contact to primate stool								
Always	0.582	37.492	2	< 0.001	0.15	9.1	2	0.011
Do not wash hands	0.139				0.20			
Sometimes	0.279				0.65			
Handwash type after contact to primate stool								
Always with soap and water	0.676	104.94	3	< 0.001	0.467	4.8	2	0.091
Water only	0.105				0.467			
Sometimes with soap and water	0.171				0.067			
Sometimes with water only	0.048				—			

### 3.8. Hygiene and Deworming Practices in Humans and Dogs in Boabeng Fiema and Shai Hills

The analysis of hygienic and deworming practices at Boabeng Fiema and Shai Hills revealed multiple statistically significant differences among variables. In both study areas, significant proportion of the respondents indicated that their compounds were mainly soil (*p* < 0.001) compared with the other variables such lawns and cemented compound (Table 4). Furthermore, in both study areas most of respondents reported washing their hands after the toilet use, and washed their hands with soap and water. The difference between variables in these categories were statistically significant (*p* < 0.001). Again, more than half of the proportion of the respondent stated that they had no knowledge of helminths compared with those who have (*p* < 0.001) in both study areas. Also, respondents who acknowledged deworming their dogs and self‐deworming were lower compared with those who did not in both Boabeng Fiema and Shai Hills (*p* < 0.001).

**Table 4:** Knowledge, hygiene, and deworming practices among humans and dogs in Boabeng Fiema and Shai Hills.

Variable	Boabeng‐Fiema Monkey Sanctuary				Shai Hills Resource Reserve			
	Proportions	Chi‐square	df	*p*value	Proportions	Chi‐square	df	*p* value
Nature of compound								
Soil	0.902	178.54	2	< 0.001	0.862	15.207	1	< 0.001
Lawn	0.008							
Cemented	0.090				0.138			
Handwash after toilet								
Always	0.943	20.759	2	< 0.001	0.724	20.759	2	< 0.001
Do not wash my hands	0.008				0.069			
Sometimes	0.049				0.207			
Handwash type								
Always with soap and water	0.793	191.96	3	< 0.001	0.741	35.27	3	< 0.001
Always with water only	0.091				0.074			
Sometimes with soap and water only	0.091				0.148			
Sometimes with water only	0.025				0.037			
Handwash before meals								
Always	0.967	105.53	1	< 0.001	0.69	4.1724	1	0.04109
Sometimes	0.033				0.31			
Handwash before meals type								
Always with soap and water	0.355	86.57	3	< 0.001	0.500	18	3	0.0004398
Always with water only	0.545				0.393			
Sometimes with soap and water only	0.058				0.036			
Sometimes with water only	0.041				0.071			
Sanitation facility								
Open defecation	0.352	2.9672	2	0.2268	0.448	10.586	3	0.01419
Private pit latrine	0.385				0.207			
Public pit latrine	0.262				0.310			
Flush toilet					0.034			
Footwear at home								
Yes	0	60.803	2	< 0.001	0	5.8276	1	0.01578
No	0.566				0.276			
Sometimes	0.434				0.724			
Dog deworm								
Never	0.705	134.72	3	< 0.001	0.905	30.857	2	< 0.001
Once every 3 months	0.090				0.048			
Once every 6 months	0.107				0.048			
Once every year	0.098				0			
Last dog deworms								
3 months ago	0.123	197.43	4	<0.001	0.105	22.211	2	<0.001
6 months ago	0.082							
Do not remember	0.066				0.053			
Never	0.705				0.842			
Once every year	0.025							
Self deworm								
Do not remember	0.180	16.689	4	0.002222	0.310	21.172	4	0.0002927
Never	0.189				0.103			
Once every 3 months	0.320				0.483			
Once every 6 months	0.221				0.034			
Once every year	0.090				0.069			
Last deworms								
3 months ago	0.238	26.033	4	< 0.001	0.414	19.103	4	0.00075
6 months ago	0.254				0.034			
Once every year	0.025				0.379			
Do not remember	0.287				0.103			
Never	0.197				0.069			
Knowledge of helminth								
Yes	0.254	29.508	1	< 0.001	0.310	4.1724	1	0.04109
No	0.745				0.689			
Knowledge of dog primate								
Yes	0.614	6.4262	1	< 0.01124	0.275	5.8276	1	0.01578
No	0.385				0.724			
Aware of possible zoonotic inf.								
Yes	0.377	7.377	1	0.006606	0.310	4.1724	1	0.04109
No	0.622				0.689			

## 4. Discussion

This study examined the prevalence and interactions of intestinal parasites among humans, dogs, and NHPs in three protected areas in Ghana: Boabeng‐Fiema Monkey Sanctuary, Mole National Park, and Shai Hills. To our knowledge, this is the first investigation in Ghana where samples were collected simultaneously from all three host populations within the same study sites. The overall prevalence recorded in human participants was 10.56% in Boabeng Fiema, 44.94% in Mole National Park, and 58.14% in Shai Hills. The comparatively low prevalence observed in Boabeng Fiema can be attributed to a mass drug administration (MDA) program in which ivermectin was distributed to the community approximately 6 months before our sample collection. The District Health Directorate confirmed this information after we recorded the low infection rates. In addition, improved hygiene practices within the community contributed to the reduced burden of intestinal parasites (pers. comm Nkoranza North Health Director). Studies conducted by Martin et al. [36], Clarke et al. [37], and Gutman et al. [38] have reported similar findings where MDA programs and improved sanitation have significantly lowered prevalence rates of soil‐transmitted helminth and protozoan infections. The prevalence recorded in Mole National Park and Shai Hills were comparatively higher. This agrees with data from studies conducted in Rwanda and Ghana, which reported a prevalence of 62.7% among outpatients attending Rubingo Health Center and 54.2% among students in Avatime Dzokpe, respectively, ([39, 40]). This high prevalence seen in Shai Hills and Mole National Park could be due to the differences in the effectiveness or coverage of MDA programs in these areas, combined with poor hygiene practices (pers. comm., West Gonja District Health Director). Although the World Health Organization (WHO) has outlined its objectives to control and eliminate neglected tropical diseases (NTDs), including soil‐transmitted helminths and protozoans, by the year 2030, progress remains uneven across different regions [41].

These human intestinal parasitic infections become important when examined alongside the parasite burden in potential animal reservoirs, particularly domestic dogs and NHPs, which share the same habitats and resources with local communities.

The overall prevalence recorded in dogs at Boabeng, Mole, and Shai Hills was 89.59%, 82.14% and 27.27%, respectively, whereas the prevalence in NHPs was 60.78%, 42.85%, and 47.72%, respectively. These high prevalences are consistent with studies conducted in similar ecological settings where dogs and NHPs roam freely and are reported as important reservoirs for potential zoonotic IPI [21, 34, 42–45]. These high prevalences seen in the dogs and NHPs are indeed alarming. This is because they frequently contaminate soil, water sources, and shared environments with parasite eggs and larvae, creating an avenue of transmission to nearby humans, potentially leading to infections, gastrointestinal diseases, and long‐term health challenges.

Following these prevalence patterns, it is also important to consider species richness. This is because species richness directly influences transmission dynamics and the potential risk to the human population through changes in parasite–host interactions [46, 47]. When considering all parasites identified across humans, dogs, and NHPs, Mole National Park recorded the highest species richness with 10 parasites detected, followed by Shai Hills with nine and Boabeng Fiema with seven. The species identified across all study areas were hookworms, *Ascaris* sp. *Strongylids* sp, *Balantidium* sp., *Diphyllobothrium* sp., *Entamoeba* sp., *Schistosoma* sp., *Trichuris* sp., *Hymenolepis* sp., *Taenia* sp., and an unknown egg. The variations in species richness observed in these areas could be a result of environmental factors (e.g., water sources, vegetation cover, and soil type) and population size of humans, dogs, and NHPs [45, 48]. Population size is considered an important factor because Shai Osudoku and West Gonja districts where Shai Hills and Mole Nationa Park are, respectively, located, have population size of 105,610 and 63,449 compared with a population of 56,468 in Nkoranza North district where Boabeng Fiema is located, respectively [49]. This study support that a larger population provides more individuals for parasites to infect, allowing a wider range of parasite species to establish and maintain the transmission cycle [48].

Among the parasites identified, hookworms and ascarids were the common parasites among humans, dogs, and NHPs in all study areas, similar to findings by Kajero et al. [50]. This observation could be interpreted as a result of social and environmental factors that create a suitable condition for cross‐species transmission of these helminths [50, 51]. Hookworms and *Ascaris* possess life cycle adaptations that enable them to persist in the environment and have broad host infectivity. *Ascaris* eggs survive in the environment for months to years in moist, shaded soil, which acts as a long‐term reservoir for reinfection. In contrast, hookworms can persist in a suitable environment for several weeks and rapidly infect any susceptible host that comes into contact with contaminated soil [51–53]. Furthermore, these parasites have a broad host range, which further explains their frequent sharing. Studies have shown that *Ancylostoma ceylanicum* and *Ascaris lumbricoides* can infect dogs, humans, and NHPs in areas where these species interact closely [54, 55]. In addition, reinfection dynamics following treatment with ivermectin, used to control these helminths, can also explain the lack of recorded *Strongyloides* sp. eggs or larvae. The hookworm reinfection can occur relatively rapidly, often noticeable within 2–4 months posttreatment, especially in endemic settings with ongoing environmental contamination [56]. In contrast, reinfection with *Strongyloides* after ivermectin treatment is less common if patients avoid re‐exposure [57]. It should also be noted that the diagnostic methodology used in this study is not really sensitive for *Strongyloides* spp. [58]. In a parallel study [28] using different methods, we detected small numbers of *Strongyloides* spp. in all three hosts, demonstrating that this genus of parasites is not fully absent from our study areas.

The questionnaire analysis gave a better understanding of the kind of interaction that happens in the various study areas. The questionnaire could not be used in Larabanga and Mognri, which are close to the Mole National Park, due to a language barrier. The questionnaire covered dog ownership and management, interactions that occur between humans and animals, hygienic practices, and knowledge of intestinal parasitic infections. Regarding ownership of dogs in both communities, 100% of the people interviewed in Boabeng Fiema own dogs, compared with 20.7% of the people in Shai Hills who own dogs. Even though only a handful of the people interviewed in Shai Hills own dogs, many stray dogs were seen in the community. High dog ownership and stray dogs′ levels can facilitate cross‐species transmission of parasites through a shared environment with humans. In addition, in Boabeng, 73.6% of the dogs were not confined, dogs roaming freely in the community and at the edge of the forest have greater exposure to wildlife feces, contaminated soil, and water sources [59]. These dogs also defecate in the communal areas, increasing transmission risk [60]. In Shai Hills, 50% of the people who own dogs semiconfine them. Again, in Boabeng, most of the dogs were fed on the floor, feeding dogs directly on the floor exposes them to various types of intestinal parasites, especially when the soil is contaminated with eggs, larvae, and cysts of these parasites.

Our findings revealed significant behavioral and environmental factors that promote the potential transmission of intestinal parasites between humans and NHPs at Boabeng and Shai Hills. The statistical analysis showed a strong association for most of the variables. Primate proximity and direct contact were strongly associated with potential exposure at both areas. These results are consistent with studies that link spatial overlap and direct human–NHP contact to increased prevalence of zoonotic intestinal parasites [61, 62]. In addition, seasonality influences exposure risk with significant associations during dry seasons at Boabeng Fiema. This could result from increased resource sharing (water sources) or a change in the ranging pattern during dry months, leading to higher encounter rates between humans and primates.

More so, environmental, behavioral, and knowledge‐related variables at Boabeng Fiema and Shai Hills revealed significant differences and risk factors of intestinal parasite transmission. In both study sites, most households had a soil compound (90.2% in Boabeng and 86.2% at Shai Hills). This is something to be taken into consideration when eliminating soil‐transmitted helminths, because soil floors serve as a suitable environment for helminth eggs and larvae, increasing exposure risk [63, 64]. These results suggest that environmental contamination could be a significant factor in the transmission of IPI in these study sites. Most respondents in both study sites practice handwashing after using the toilet and before meals. Open defecation is still rather common (49% of the respondents in Shai Hills and 34% in Boabeng), likely leading to environmental contamination. In both study sites, many respondents were inconsistent in wearing footwear at home, a known risk for exposure to hookworm and *Strongyloides* sp. Self‐deworming and dog‐deworming practices were poor across study sites, with a large majority of dog owners never deworming their dogs, which also promotes the likelihood of environmental contamination. Knowledge on helminths and zoonotic diseases was low in these areas, suggesting the need to educate people in similar settings to our study areas.

Although most people across the study sites practice proper handwashing, it is important to consider the gaps in footwear use, deworming, sanitation practices, and awareness of intestinal parasitic infections. These factors are known to be major contributing factors to ongoing transmission risk [65, 66]. It is therefore important to integrate interventions addressing these factors to control intestinal parasitic infection in these communities effectively.

## 5. Limitations of Our Study

This study focused exclusively on households living with dogs (either owning or being exposed to stray dogs). Hence, the findings may not reflect the full picture of the population in the study area. In addition, some community members were reluctant to participate, which resulted in a relatively small sample size in some of the communities and possibly a bias in responses because we cannot exclude that there is a correlation between certain practices and the willingness to participate in the study. In addition, to process more samples, we limited ourselves to a single diagnostic method, which is not equally sensitive to different parasites.

## 6. Conclusion

Our study is the first comprehensive study to simultaneously analyze intestinal parasites in humans, domestic dogs, and NHPs within three protected areas in Ghana. The findings showed differences in prevalence and species richness across the study areas. Boabeng Fiema recorded lower human infection rates linked to ivermectin administration before the study′s sample collection. The high prevalence′s recorded in dogs and NHPs and the shared parasite species with humans reveal a significant risk of cross‐species transmission. In addition, environmental contamination, open defecation, poor self‐ and dog‐deworming practices, and inadequate knowledge on intestinal parasitic infections continue to sustain the transmission cycle. Targeted One Health Interventions, combined with improved sanitation, effective treatment, community education, and managing both domestic and wild animal reservoirs, will help reduce the burden of intestinal parasitic infections.

## Funding

No funding was received for this manuscript.

## Conflicts of Interest

The authors declare no conflicts of interest.

## Data Availability

The data that support the findings of this study are available from the corresponding author upon reasonable request.
